# Caring for Coronavirus Healthcare Workers: Lessons Learned From Long-Term Monitoring of Military Peacekeepers

**DOI:** 10.3389/fpsyg.2020.566199

**Published:** 2020-10-19

**Authors:** Christer Lunde Gjerstad, Hans Jakob Bøe, Erik Falkum, Andreas Espetvedt Nordstrand, Arnfinn Tønnesen, Jon Gerhard Reichelt, June Ullevoldsæter Lystad

**Affiliations:** ^1^Institute of Military Psychiatry, Norwegian Armed Forces Joint Medical Services, Oslo, Norway; ^2^Institute of Clinical Medicine, Faculty of Medicine, University of Oslo, Oslo, Norway; ^3^Division of Mental Health and Addiction, Kongsberg DPS, Vestre Viken Hospital Trust, Kongsberg, Norway; ^4^Division of Mental Health and Addiction, Department of Research, Oslo University Hospital, Oslo, Norway; ^5^Department of Psychology, Norwegian University of Science and Technology (NTNU), Trondheim, Norway; ^6^Norwegian Armed Forces Joint Medical Services, Oslo, Norway; ^7^Section of Early Psychoses Treatment, Division of Mental Health and Addiction, Oslo University Hospital, Oslo, Norway

**Keywords:** COVID-19, mental health, posttraumatic stress disorder, resilience (psychological), healthcare workers, military

## Abstract

**Background:**

The current outbreak of the coronavirus disease (COVID-19) is of unprecedented proportions in several regards. Recent reports suggest that many frontline healthcare workers (HCWs) suffer from mental health problems, including posttraumatic stress symptoms (PTSS). Previous studies have identified several key factors associated with short-term PTSS in pandemic HCWs, yet limited data is available on factors associated with long-term PTSS. Understanding the psychological impact of the pandemic on HCWs is important in planning for future outbreaks of emerging infectious diseases. In the current study, we look to findings from a highly relevant subsection of the trauma field, the military domain.

**Objective:**

Pandemic HCWs and military peacekeepers may experience similar stressors in the line of duty. This study investigated whether factors linked to short-term PTSS in pandemic HCWs were also associated with long-term PTSS in military peacekeepers.

**Materials and Methods:**

Peacekeepers who reported pandemic-relevant stressors during deployment to a UN peacekeeping mission were included in the study (*N* = 1,627). PTSS was self-reported using the Posttraumatic Stress Disorder Checklist – Military Version. Descriptive instruments were used to assess possible factors associated with PTSS. A multiple linear regression analysis was performed to explore associations between these factors and PTSS.

**Results:**

Our model accounted for 50% of the variance in PTSS, *F*(1503,11) = 139.00, *p* < 0.001. Age, relationship and employment status, preparedness, working environment, social support after deployment, barriers to disclose, recognition, and loneliness were all significantly associated with PTSS on average 30 years after deployment. The most important risk factors of long-term PTSS were personal barriers to disclose one’s experiences and current unemployment.

**Conclusion:**

Several factors linked to short-term PTSS in pandemic HCWs were associated with long-term PTSS in peacekeepers. We discuss how these findings may be used to prevent long-term PTSS in HCWs involved in the current COVID-19 outbreak.

## Introduction

At time of writing, the world is struggling to cope with a coronavirus disease (COVID-19) pandemic of unprecedented proportions. As of August 20th, 2020, the World Health Organization (2020) has estimated almost 20 million confirmed cases and nearly 780,000 confirmed deaths in 216 countries. In this regard, many have voiced concern over the potential burden that is being put upon healthcare workers (HCWs) involved in the treatment of COVID-19 patients (i.e., Greenberg et al., 2020; Truog et al., 2020). Due to rapidly growing numbers of critically ill patients, no approved vaccine, and shortages of essential medical resources and staff, these HCWs are currently dealing with serious challenges (Chen et al., 2020). Some have even gone as far as comparing the current trials of HCWs to those of war (Horton, 2020). In particular, long and irregular work hours, shortages of personal protective equipment (PPE), personal infection risk, fear of infecting friends and family, social isolation, moral dilemmas such as deciding who to prioritize for life-saving treatments, and feelings of helplessness when not being able to help dying patients may be the brutal reality for many HCWs involved in the COVID-19 effort (Kang et al., 2020).

Research following previous pandemics like the 2003 severe acute respiratory syndrome (SARS) outbreak has shown that stressors experienced by HCWs may pose an imminent risk of stress reactions and development of adverse mental health consequences (Bai et al., 2004; Chua et al., 2004; Lee et al., 2007). In line with this, recent data from hospital wards involved in the treatment of COVID-19 patients indicate that a substantial number of HCWs experience symptoms of mental health problems, such as depression, anxiety, insomnia, and posttraumatic stress disorder (PTSD; Huang et al., 2020; Lai et al., 2020; Spoorthy, 2020).

In an effort to aid the world in this time of need, prominent voices within the trauma research community have encouraged researchers to “employ all heavy guns of scientific practice, including novel statistical analysis, unique study designs, and creative collaborations and combinations of trauma disciplines in order to deepen our understanding of the mental health implications of the current crisis” (Horesh and Brown, 2020). Attempting to respond to this call, we look to findings from a relevant subsection of the trauma field, namely, the military domain. Although not directly comparable, the challenges and dilemmas soldiers face during military peacekeeping missions are similar in nature to the current struggles of HCWs (Greenberg et al., 2020).

Peacekeeping personnel routinely make quick life-or-death decisions based on uncertain information, as well as working for extended periods in hazardous, high-risk environments with elevated levels of psychological stress. Moreover, peacekeepers are often unable to intervene due to mandate restrictions when witnessing suffering in other, often innocent parties. Thus, they frequently deal with experiences of helplessness. In addition, peacekeeping personnel and HCWs share the common feature that they are, to some extent, trained and prepared for an exceptional work environment. Finally, deployment as a peacekeeper involves enduring long-time separation from family and loved ones (Weisæth and Sund, 1982; Mehlum and Weisæth, 2002; Norwegian Armed Forces Joint Medical Services, 2016). This experience may be comparable to the self-isolation many pandemic HCWs impose on themselves to reduce risk of infecting loved ones.

Given that stressors experienced by military peacekeepers and COVID-19 HCWs are similar, lessons learned from past peacekeeping missions may have potential to inform today’s situation. We know from previous pandemic research that several factors influence levels of psychological distress among HCWs. In particular, prior training and preparedness, workload levels, opportunities for rest and recuperation, social support, personal barriers to disclose one’s experiences, recognition and acknowledgment, and feelings of loneliness have been found to impact stress levels (Maunder et al., 2003; Chan and Huak, 2004; Tam et al., 2004; Marjanovic et al., 2007; Khalid et al., 2016; Brooks et al., 2018; Huremović, 2019; Chen et al., 2020; Xiao et al., 2020). However, most of these studies have only examined mental health consequences during or shortly after a pandemic; little is known of what predicts mental health over time. Data on long-term mental health consequences following high stressor exposure is available in military populations (i.e., Gjerstad et al., 2020). Applying these data to identify factors important for long-term mental health outcomes may help recognize possible areas of support and intervention for HCWs facing today’s challenges. By looking at subgroups of military peacekeepers reporting stressors akin to those of HCWs involved in the COVID-19 effort, we may find characteristics of those who retain their mental health despite deeply challenging circumstances. Such knowledge may be of use to hospitals in supporting critical frontline personnel and preventing adverse mental health consequences in the long run.

In the current study, we examined data from a large, post-deployment survey of soldiers deployed to a UN peacekeeping operation. Specifically, we wanted to explore whether factors that have been linked to short-term stress and mental health problems among pandemic HCWs are also associated with long-term posttraumatic stress symptoms (PTSS) in peacekeepers reporting similar stressor exposure during deployment. Hopefully, these findings may be transferable to the civilian healthcare domain and prove valuable in caring for HCWs in the years following this pandemic.

## Materials and Methods

### Participants

The study used data from a cross-sectional, post-deployment survey of Norwegian peacekeepers deployed to the United Nations Interim Force in Lebanon (UNIFIL). All Norwegian military personnel deployed to Lebanon between 1978 and 1998 were invited to participate, in total 20,678 men and women. Of the invited personnel, 11,633 responded. However, 1,028 of these were either active refusals (913) or incomplete responses (115), resulting in 10,605 valid responses and a final positive response rate of 51.3%. The response rate was comparable to those obtained in other studies on military populations (i.e., McAndrew et al., 2013; Forbes et al., 2016).

A comparison of the demographic characteristics of responders and non-responders showed that responders were slightly older and had lower frequencies of sick leaves and benefits. A complete description of the demographic characteristics of responders and non-responders have been published elsewhere (Gjerstad et al., 2020).

For the current study, peacekeepers who reported pandemic-relevant stressors during deployment (*N* = 1,627) were identified and included in the final sample for further analyses. Pandemic-relevant stressors were defined as stressors similar to the ones experienced by HCWs during pandemics (Greenberg et al., 2020), such as providing care to critically wounded people, being exposed to dangerous or toxic environments, risking infection from serious illnesses, making mistakes/misjudgments that result in harm or death to others, participating in morally questionable actions, or failing to take action when deemed necessary. Relevant items were discussed in the research group, achieving consensus on constructs reflecting the research objective. See Supplementary Appendix A for a complete list of items. Only peacekeepers who reported at least one pandemic-relevant stressor *and* rated the stressor as moderately/extremely stressful were included. Mean time since deployment in the sample was 30 years (range: 18–38 years).

Sex and age group (in years: 30–39, 40–49, 50–59, 60–69, 70+) were extracted from the Norwegian Labor and Welfare Administration (NAV). Current relationship status (in a relationship, single) and employment status (employed, unemployed) were self-reported by the respondents at the time of survey. See Table 1 for demographic characteristics of the sample.

**TABLE 1:** Demographic characteristics of the study population (*N* = 1,627).

**Characteristic**	***N***	***n***	***%***
Sex	1627		
Female		23	1.4
Male		1604	98.6
Age Group, Years	1627		
30–39		9	0.6
40–49		397	24.4
50–59		849	52.2
60–69		312	19.2
70+		60	3.7
Relationship Status	1612		
In a Relationship		1191	73.9
Single		421	26.1
Employment Status	1568		
Employed		1196	76.3
Unemployed		372	23.7

### Procedure

A printed version of the survey questionnaire, as well as a letter containing an internet link and unique login credentials, were mailed to all invited participants, giving them the choice of answering either the printed version or an equivalent digital version of the questionnaire. The data collection phase lasted from September 2014 to April 2015 and included two reminders.

### Measures

#### Dependent Variable

##### Posttraumatic stress disorder checklist – military version (PCL-M)

The PCL-M (Weathers et al., 1993) is a commonly used self-rating instrument containing 17 items representing the Diagnostic and Statistical Manual of Mental Disorders, 4th ed., text rev. (DSM-IV-TR; American Psychiatric Association, 2000) diagnostic criteria for PTSD. It is a well-validated measure for screening of PTSS in military populations and shows good temporal stability, internal consistency, and convergent validity (Wilkins et al., 2011). Respondents were asked to rate the frequency of symptoms experienced during the past week. Each item was rated on a 5-point Likert scale with the response categories 1 (*not at all*); 2 (*a little bit*); 3 (*moderately*); 4 (*quite a bit*); and 5 (*extremely*), giving a total score range of 17–85 (*M* = 32.58, *SD* = 16.63, *SE* = 0.41, α = 0.97). A higher score indicated more PTSS.

#### Independent Variables

The instruments described in this section were constructed specifically to capture the unique experiences of Norwegian Armed Forces personnel deployed to Lebanon or Afghanistan (Norwegian Armed Forces Joint Medical Services, 2012, 2016). The instruments were mainly assembled to serve important descriptive purposes; hence, most of them were not yet empirically validated. As recommended by Eisinga et al. (2013), Cronbach’s coefficient alpha was reported as a measure of reliability for instruments containing three or more items, while Spearman–Brown coefficient was reported for instruments containing only two items.

##### Preparedness

Preparedness was measured by the following two items: “*The service corresponded to my civilian or military education or work experience*” and “*I was given adequate training and was well prepared for the service.*” Respondents were asked to indicate how much they agreed with each statement on a 5-point Likert scale with the response categories 1 (*not at all*); 2 (*to a small degree*); 3 (*to some degree*); 4 (*to a large degree*); and 5 (*to a very large degree*), giving a total score range of 2–10 (*M* = 6.06, *SD* = 1.82, *SE* = 0.05, *r*_SB_ = 0.50). A higher score indicated a higher degree of preparedness.

##### Workload

Workload was measured by the following two items: “*The workload was too heavy*” and “*The work was demanding.*” Respondents were asked to indicate how much they agreed with each statement on a 5-point Likert scale with the response categories 1 (*not at all*); 2 (*to a small degree*); 3 (*to some degree*); 4 (*to a large degree*); and 5 (*to a very large degree*), giving a total score range of 2–10 (*M* = 6.73, *SD* = 1.68, *SE* = 0.04, *r*_SB_ = 0.67). A higher score indicated a higher workload.

##### Rest and recuperation

Rest and recuperation were measured by five statements concerning opportunities for rest/sleep, recreation, and personal space, as well as sanitary conditions and access to food/drink during deployment. Respondents were asked to indicate how much they agreed with each statement on a 5-point Likert scale with the response categories 1 (*not at all*); 2 (*to a small degree*); 3 (*to some degree*); 4 (*to a large degree*); and 5 (*to a very large degree*), giving a total score range of 5–25 (*M* = 14.96, *SD* = 3.65, *SE* = 0.09, α = 0.81). A higher score indicated better opportunities for rest and recuperation.

##### Social support

Social support was measured both as perceived support from colleagues and superiors during deployment and as perceived access to social support after deployment.

Social support during deployment was gauged by the following two items: “*I experienced cohesion and support from my colleagues*” and “*I had superiors who were supportive of me.*” Respondents were asked to indicate how much they agreed with each statement on a 5-point Likert scale with the response categories 1 (*not at all*); 2 (*to a small degree*); 3 (*to some degree*); 4 (*to a large degree*); and 5 (*to a very large degree*), giving a total score range of 2–10 (*M* = 6.94, *SD* = 1.66, *SE* = 0.04, *r*_SB_ = 0.55). A higher score indicated a higher degree of social support.

Social support after deployment was gauged by the following two items: “*In the time after deployment, I had access to people who could support me if I had problems*” and “*In the time after deployment, how many people were so close to you that you could count on them for support if you had substantial personal problems?.*” Respondents were asked to indicate how much they agreed/how many close confidents they had on a 5-point Likert scale with the response categories 1 (*not at all/none*); 2 (*to a small degree/1 person*); 3 (*to some degree/2 persons*); 4 (*to a large degree/3*–*5 persons*); and 5 (*to a very large degree/6+ persons*), giving a total score range of 2–10 (*M* = 6.47, *SD* = 2.31, *SE* = 0.06, *r*_SB_ = 0.68). A higher score indicated a higher degree of social support.

##### Personal barriers to disclose

The measure of personal barriers to disclose one’s experiences was developed by the project group for the 2012 Afghanistan Study (Norwegian Armed Forces Joint Medical Services, 2012; Nordstrand et al., 2020). Respondents were asked to relate to their deployment and rate the following three items: “*I experienced incidents which I have not been able to tell others about, not even those closest to me*”; “*I have had/have problems that I am not able to share with family or friends*”; “*There is no one at home who is able to understand what I have experienced.*” Each item had a 5-point Likert response format with the following response categories: 1 (*completely disagree*); 2 (*disagree somewhat*); 3 (*either/or*); 4 (*agree somewhat*); and 5 (*completely agree*), giving a total score range of 3–15 (*M* = 8.31, *SD* = 3.52, *SE* = 0.09, α = 0.76). A higher score indicated more personal barriers to disclose.

##### Recognition

Recognition was measured by five statements concerning perceived recognition and acknowledgment of one’s effort by government/politicians, media/public debate, family/friends, society in general, and the armed forces. Each item had a 5-point Likert response format with the following response categories: 1 (*completely disagree*); 2 (*somewhat disagree*); 3 (*either/or*); 4 (*somewhat agree*); and 5 (*completely agree*), giving a total score range of 5–25 (*M* = 14.39, *SD* = 4.49, *SE* = 0.11, α = 0.87). A higher score indicated a higher degree of recognition.

##### Loneliness

Loneliness was measured by a single item: “I felt lonely.” Respondents were asked to indicate how much they agreed with the statement on a 5-point Likert scale with the response categories 1 (*not at all*); 2 (*to a small degree*); 3 (*to some degree*); 4 (*to a large degree*); and 5 (*to a very large degree*), giving a total score range of 1–5 (*M* = 2.02, *SD* = 0.93, *SE* = 0.02). A higher score indicated a stronger feeling of isolation/loneliness.

### Data Analysis

Descriptive statistics were used to report demographic characteristics. A correlation matrix displayed bivariate relationships between the regression variables. Multiple linear regression analysis was executed to explore key factors associated with PTSS. All variables were entered in the same step. The tests of collinearity (i.e., tolerance and VIF) were all within acceptable limits (Hair et al., 2014). In cases of missing data, listwise deletion was employed. This applied for up to 3.6% of the sample. All analyses were performed using IBM SPSS Statistics version 25.0 (IBM Corp, 2017).

## Results

Age, relationship status, and employment status have previously been identified as potential confounder variables in trauma studies (Chan and Huak, 2004; Tam et al., 2004; Bosmans and Der Velden, 2018). Hence, they were included as control variables in the regression analysis. Due to the large sex bias in the current sample (98.6% males), we did not control for sex. See Table 1 for demographic characteristics of the sample.

The intercorrelation matrix showed significant small to medium correlations between all independent variables (except age) and PTSS, with the strongest correlations being with personal barriers to disclose (*r* = 0.54) and social support after deployment (*r* = −0.45). There were also significant small to medium correlations between several of the independent variables. See Table 2 for complete intercorrelation matrix.

**TABLE 2:** Intercorrelation matrix (Pearson two-tailed) for PTSS and independent variables (*N* = 1,627).

	***M***	***SD***	**1**	**2**	**3**	**4**	**5**	**6**	**7**	**8**	**9**	**10**	**11**	**12**
1. PTSS	32.58	16.63												
2. Age Group	4.01	0.78	–0.01											
3. Relationship Status			0.21***	0.07**										
4. Employment Status			0.31***	0.30***	0.25***									
5. Preparedness	6.06	1.82	−0.29***	0.02	–0.04	–0.05								
6. Workload	6.73	1.68	0.29***	0.05*	0.03	0.11***	−0.14***							
7. Rest and Recuperation	14.96	3.65	−0.37***	−0.11***	−0.05*	−0.10***	0.41***	−0.43***						
8. Social Support During	6.94	1.66	−0.22***	−0.07**	−0.06*	−0.08**	0.24***	–0.02	0.26***					
9. Social Support After	6.47	2.31	−0.45***	–0.05	−0.12***	−0.19***	0.25***	−0.14***	0.27***	0.28***				
10. Barriers to Disclose	8.31	3.52	0.54***	−0.06*	0.07**	0.16***	−0.25***	0.31***	−0.35***	−0.17***	−0.44***			
11. Recognition	14.39	4.49	−0.39***	0.11***	−0.07**	−0.09***	0.29***	−0.16***	0.30***	0.27***	0.37***	−0.39***		
12. Loneliness	2.02	0.93	0.39***	–0.03	0.09**	0.13***	−0.25***	0.19***	−0.31***	−0.43***	−0.31***	0.28***	−0.25***	

Spearman’s rho is reported for correlations involving the two dichotomous variables relationship status and employment status. * *p* < 0.05, ** *p* < 0.01, *** *p* < 0.001.

The results of the regression analysis are displayed in Table 3. Overall, the model accounted for 50% of the variance in PTSS, *F*(1503,11) = 139.00, *p* < 0.001, and all variables except social support during deployment were significantly associated with PTSS. Lower age, being single, and being unemployed at the time of survey were associated with more PTSS. Being unemployed at the time of survey was the most important factor among the demographic variables (*β* = 0.26, *p* < 0.001). In terms of the other independent variables, a higher degree of preparedness, better opportunities for rest and recuperation, more social support after deployment, and more perceived recognition were associated with less PTSS, while higher workload, more personal barriers to disclose one’s experiences, and a stronger feeling of loneliness were associated with more PTSS. Personal barriers to disclose was the single most important factor associated with PTSS (*β* = 0.29, *p* < 0.001).

**TABLE 3:** Summary of multiple linear regression analysis for factors associated with PTSS (*N* = 1,514).

	***B***	***SE B***	***β***
Demographics			
Age Group	–1.73	0.43	−0.08***
Relationship Status	3.49	0.72	0.09***
Employment Status	10.33	0.80	0.26***
Preparedness	–0.52	0.19	−0.06**
Working Environment			
Workload	0.61	0.21	0.06**
Rest and Recuperation	–0.41	0.11	−0.09***
Social Support			
During	0.09	0.21	0.01
After	–1.02	0.16	−0.14***
Barriers to Disclose	1.37	0.10	0.29***
Recognition	–0.39	0.08	−0.10***
Loneliness	2.30	0.38	0.13***
Adj. *R*^2^		0.50	
*F*		139.00***	

** *p* < 0.01, *** *p* < 0.001.

## Discussion

### Summary of Main Findings

Our regression model showed that age, relationship and employment status, preparedness, working environment, social support after deployment, barriers to disclose, recognition, and loneliness were all significantly associated with long-term PTSS in our sample of peacekeepers. Social support during deployment was, however, not associated with PTSS. The most important risk factors of PTSS were personal barriers to disclose one’s experiences and unemployment at time of survey.

### Demographic Variables

Lower age, being single, and being unemployed were all risk factors of long-term PTSS, with unemployed being most important. The relationship between unemployment and mental health problems has been confirmed through review studies (i.e., Shuo and Vishal, 2013). An explanation of the adverse consequences of unemployment may be found in the so-called *healthy worker effect phenomenon*; employed individuals tend to have lower morbidity and mortality rates than unemployed individuals (Shah, 2009). Recent findings suggest that this effect is also relevant in a post-trauma recovery context, where employment is associated with significantly lower levels of posttraumatic stress (Bosmans and Der Velden, 2018). Further, preliminary results from a study investigating factors associated with mental health problems in the general public during the COVID-19 pandemic suggest that employment protects against mental health problems (Ebrahimi et al., 2020). Healthcare workers’ employment status in the aftermath of a pandemic should thus be considered, as unemployment could prolong the process of recovery and lead to more severe posttraumatic stress reactions over time. Particular attention should be paid to those HCWs who have been recruited specifically to work with COVID-19 patients due to extraordinary staffing needs (i.e., Mansoor, 2020). Such HCWs may be students, retired or otherwise outside the workforce, and possibly be more likely to experience unemployment after the pandemic has passed.

### Preparedness

A higher degree of preparedness, in terms of sufficient training and correspondence between previous education/work experience and service, was associated with fewer symptoms of long-term posttraumatic stress in our sample. Preparedness may be a key factor in the development of PTSS by means of its association with perceived threat (Schnurr et al., 1993). In the case of the current pandemic, realistic training and preparations will likely reduce stress levels and perceptions of threat among frontline HCWs and hence mitigate development of long-term PTSS (Greenberg et al., 2015; Tan et al., 2020). Preparedness may also protect HCWs from feeling overwhelmed and increase their ability to maintain the professional stance and distance needed for coping with the pandemic over time. Specifically, these findings might highlight the importance of allotting time for HCWs to familiarize themselves with novel medical procedures and practicing technical skills.

### Working Environment

Workload and rest and recuperation were both significantly associated with PTSS. Higher workload was associated with more PTSS, while better opportunities for rest and recuperation were associated with less PTSS. This is concurrent with previous findings (Litz, 2014; Prince et al., 2015; Chappelle et al., 2019) and is easily transferrable to the ongoing pandemic. High workloads and excessive work hours have been highlighted as potential sources of mental health problems in COVID-19 HCWs (Spoorthy, 2020). From the field of occupational medicine, workload and shift duty are well-known workplace stressors (McFarlane and Bryant, 2007), and in the context of a pandemic, this is adding to potentially traumatic experiences. Optimally, hospitals and healthcare services should identify and manage workload risks at an organizational level, avoiding adverse consequences in a timely manner. Managing such risks also entails facilitating sufficient opportunities for rest and recuperation for HCWs. Moreover, if possible, ensuring that taxing work assignments are rotated between personnel may be an important stress-preventive strategy (Marjanovic et al., 2007; Adriaenssens et al., 2015). Potential pitfalls may otherwise be non-attendance due to stress, excessive workload, prospective illness, and long-term mental health problems.

### Social Support and Personal Barriers to Disclose

Perceived social support from colleagues and superiors during deployment was not significantly associated with long-term PTSS. This contrasts with findings from the healthcare domain. Several studies have documented that social support in the workplace is negatively associated with general psychiatric symptoms and PTSS in pandemic HCWs (Chan and Huak, 2004; Tam et al., 2004). Previous studies have hypothesized that the links between social support and PTSS may be dependent upon trauma typology (Valentiner et al., 1996; Ullman and Filipas, 2001). In particular, the moderating effects of social support on morally challenging traumas may be sensitive to both the type of social support given and from whom the social support is provided. In other words, the impact of social support may be greater if it is provided by close friends or significant others, especially if the relevant trauma is morally challenging. A characteristic of the stressor exposure of both peacekeepers and HCWs is the common occurrence of morally challenging traumas (Jordan et al., 2017; Kang et al., 2020).

The protective effect of post-trauma social support is documented in several studies on both military and civilian populations. In a recent study, Nordstrand et al. (2020) examined the effect of post-trauma social support on posttraumatic development in a sample of Afghanistan veterans. The authors also looked at how social support interacted with personal barriers to disclose traumatic experiences. Although barriers were originally associated with posttraumatic deprecation, this effect diminished when social support was included in the model. The authors concluded that post-trauma social support seemed to buffer against the negative effect of barriers; however, this effect will have to be confirmed by prospective studies.

In the current study, a similar negative association was found between barriers to disclose and PTSS. In fact, barriers to disclose was the most important factor associated with PTSS in our regression model. However, unlike in Nordstrand and colleagues’ model (Nordstrand et al., 2020), both social support and barriers to disclose were significantly associated with PTSS, albeit in opposite directions. Although perceived social support in the aftermath of trauma seems to protect against long-term PTSS, barriers to disclose may weaken this effect. Hence, it is vital to overcome personal barriers to disclose one’s experiences in order to utilize available social support. This is concurrent with findings from both the military and civilian trauma domain (Ullman and Filipas, 2001; Guay et al., 2006; Thoresen et al., 2014).

Moreover, studies have demonstrated strong correlations between morally challenging traumas and socially inhibitory feelings such as guilt and shame (Ramage et al., 2016; Jordan et al., 2017; Nordstrand et al., 2019), thus increasing reticence to talk about such experiences (Pietrzak et al., 2009; Gray et al., 2012). Accordingly, it may be important to not only be aware of the potentially morally challenging stressors HCWs face but also help lower barriers toward disclosing such stressors to significant others. Our findings further imply that healthcare administrators should facilitate mechanisms and support systems that help break down such barriers and encourage HCWs to share their experiences. Removing barriers to disclose seems crucial to prevent long-term PTSS.

### Recognition

Perceived recognition of effort was significantly associated with lower levels of long-term PTSS in our sample. Recognition in the form of positive homecoming receptions and similar appreciative events have previously been associated with less psychological distress in peacekeepers (Sareen et al., 2010). Similarly, a study investigating organizational support to HCWs during the Toronto SARS outbreak found that recognition from hospital management was associated with lower perceived personal threat and less emotional exhaustion (Fiksenbaum et al., 2006). Further, a lack of positive media coverage, albeit a more circumferential measure of public acknowledgment, has been found to impact mental health in peacekeepers negatively, leading them to feel forgotten and less important (Raju, 2014). Societal recognition and acknowledgment are thus factors likely to be related to mental health, both in peacekeepers and in pandemic HCWs. Sufficient public support and recognition may consequently be key determinants of post-outbreak mental health in COVID-19 HCWs.

### Loneliness

Although only measured with a single item, loneliness had a strong positive association with PTSS in the current study. Peacekeepers who reported feeling lonely during deployment also reported more long-term symptoms of PTSS. Loneliness is not unique to peacekeeping or military personnel; HCWs may be confronted with similar circumstances during the ongoing pandemic. Whereas most people are encouraged to work from and stay at home with their families, HCWs face higher workloads, working in shifts as well as having to deal with serious illness. This, combined with a concern of potentially infecting friends and family, may result in social isolation and, in turn, loneliness (Ornell et al., 2020).

Loneliness is meanwhile a well-established associate of poor physical health (Hawkley et al., 2010; Valtorta et al., 2016). However, loneliness is also a prominent risk factor of mental illness (Masi et al., 2011; Wang et al., 2018). A meta-analysis by Masi et al. (2011) identified strategies such as enhancing social support and increasing possibilities for social interactions as important interventions for reducing loneliness. Applied to the ongoing pandemic, providing HCWs with increased access to activities considered to be effective coping mechanisms (Shwalb, 2007) may be of importance. Further, the use of digital platforms to maintain contact with close ones may also prevent loneliness to a certain degree (Chen et al., 2020). Finally, formal and informal social support from managers and coworkers in terms of improving open communication and establishing buddy systems for collegial support may be important preventive measures (de Boer et al., 2014).

### Limitations

Several methodological issues warrant consideration. The cross-sectional nature of the study does not allow for causal interpretation of the data; longitudinal studies are needed to explore temporal relationships between the independent variables and PTSS. Further, it could be argued that surveying respondents about what they experienced on average 30 years ago makes the data vulnerable to recollection bias. However, studies have demonstrated that the fear of recollection bias is often exaggerated (McNally, 2003). Moreover, self-report may be viewed as an unreliable way of measuring posttraumatic stress. Bearing this in mind, we have used symptoms of posttraumatic stress rather than cases of PTSD as the dependent variable.

To accommodate requirements of brevity and applicability to the research setting and the population, some of the independent variables were measured using unvalidated questionnaires. The reliance on these measures’ face validity may be a limitation that should be considered.

Finally, caution should be applied in generalizing these results from peacekeepers to pandemic HCWs. Although peacekeepers and HCWs face similar stressors during service, a peacekeeping mission and a pandemic are ultimately two different things. In addition, most peacekeepers in our sample were male, whereas most HCWs are female (Boniol et al., 2019). However, *post hoc* analyses revealed that the PTSS distributions were not significantly different between male and female peacekeepers, *t*(1603) = −0.89, *p* = 0.37. Further, sex was not significantly associated with PTSS when added to the regression model (*β* = 0.00, *p* = 0.815). Thus, our findings may hopefully be of relevance to both male and female frontline personnel exposed to major stressors or potentially traumatic events in the line of duty.

### Conclusion and Clinical Implications

The current study has identified several key factors associated with long-term posttraumatic stress in a sample of military peacekeepers exposed to pandemic-relevant stressors. Our results seem to confirm that factors linked to short-term stress and mental health problems among pandemic HCWs are also associated with long-term PTSS in peacekeepers. These findings may transfer to HCWs facing the ongoing coronavirus pandemic. Importantly, we suggest that healthcare administrators facilitate social support systems that encourage HCWs to share difficult experiences with others, as this may prevent feelings of loneliness and help maintain resilience in the face of crisis. Moreover, particular attention should be paid to HCWs who have been recruited specifically to work with coronavirus patients due to extraordinary staffing needs, but who are usually either students, retired or otherwise outside the workforce. These individuals may be less prepared and more at risk of PTSS. Attending to these concerns may prove valuable in alleviating long-term mental health problems in this all-important group of frontline health care providers.

## Data Availability Statement

The datasets presented in this article are not readily available because they are stored in accordance with the existing legislation regulating the Norwegian Armed Forces Health Registry. Requests to access the datasets should be directed to the Norwegian Armed Forces Health Registry, email: datatilgang@forsvaretshelseregister.no.

## Ethics Statement

The studies involving human participants were reviewed and approved by the Norwegian Armed Forces Joint Medical Services Research Council and the Regional Committee for Medical and Health Research Ethics of South-East Norway. The patients/participants provided their written informed consent to participate in this study.

## Author Contributions

CG and JL contributed to the theoretical conception of the study and the interpretation of the results, performed the statistical analyses, and drafted the manuscript. All authors reviewed and edited the manuscript and made a substantial intellectual contribution to the manuscript and approved it for publication.

## Conflict of Interest

The authors declare that the research was conducted in the absence of any commercial or financial relationships that could be construed as a potential conflict of interest.
