# Supplementary material for: Caring for Coronavirus Healthcare Workers: Lessons Learned From Long-Term Monitoring of Military Peacekeepers
Source: Front Psychol. 2020 Oct 19;11:566199. doi: 10.3389/fpsyg.2020.566199 (PMC7604419; doi:10.3389/fpsyg.2020.566199)
Supplement: Supplementary file 1 [file Table_1.DOCX]

# **Appendix A**

Pandemic-Relevant Stressors

Please indicate if you experienced any of the following during deployment.

If you answer “Yes”, please indicate how stressful the experience was to you.

|  | **Did you experience any of the following?** | | | | **If “Yes”, how stressful was the experience?** | | | |
| --- | --- | --- | --- | --- | --- | --- | --- | --- |
| **Item** | **No** | **Yes,  1-2 times** | **Yes,  3-5 times** | **Yes,  5+ times** | **Not at all** | **Mildly** | **Moderately** | **Extremely** |
| 1. I provided first aid to seriously injured individuals |  |  |  |  |  |  |  |  |
| 1. I walked or drove through a landmine area/IED area not cleared of mines |  |  |  |  |  |  |  |  |
| 1. I was exposed to harmful smoke, strongly polluted air/drinking water or other environmental toxins |  |  |  |  |  |  |  |  |
| 1. I was infected with malaria, yellow fever or other serious ailments |  |  |  |  |  |  |  |  |
| 1. Did you ever come to harm fellow soldiers or other team members by accident? |  |  |  |  |  |  |  |  |
| 1. Did you ever misjudge a situation that led to injury or the death of fellow soldiers or other team members? |  |  |  |  |  |  |  |  |
| 1. Did you ever misjudge a situation that lead to injury or death of civilians or enemies? |  |  |  |  |  |  |  |  |
| 1. Did you ever wound or kill someone during deployment that you in hindsight think of as unnecessary, and thus regret? |  |  |  |  |  |  |  |  |
| 1. Did you engage in morally questionable actions? |  |  |  |  |  |  |  |  |
| 1. Did you avoid taking action in situations where, with the benefit of hindsight, in which you should have? |  |  |  |  |  |  |  |  |

**Appendix B**

Rest and Recuperation

Please indicate how much you agree with the following statements.
If you have several deployments to Lebanon, please base your answers on your experiences from the most stressful deployment.

| **Item** | **Not at all** | **To a small degree** | **To some degree** | **To a large degree** | **To a very large degree** |
| --- | --- | --- | --- | --- | --- |
| 1. Opportunities for rest and recreation were satisfactory |  |  |  |  |  |
| 2. I could spend time on my own when needed |  |  |  |  |  |
| 3. I was mostly fully rested during service |  |  |  |  |  |
| 4. Sanitary conditions were satisfactory |  |  |  |  |  |
| 5. I had satisfactory access to food and water |  |  |  |  |  |

**Appendix C**

Recognition

Please indicate how much you agree with the following statements.
If you have several deployments to Lebanon, please base your answers on your combined experience from all deployments.

| **I feel that my effort has been recognized/acknowledged by…** | **Completely disagree** | **Somewhat disagree** | **Either/or** | **Somewhat agree** | **Completely agree** |
| --- | --- | --- | --- | --- | --- |
| 1. The government/politicians |  |  |  |  |  |
| 2. The media/in the public debate |  |  |  |  |  |
| 3. Family and friends |  |  |  |  |  |
| 4. Society in general |  |  |  |  |  |
| 5. The armed forces |  |  |  |  |  |
